# Editorial: Progression to Diabetes: Molecular and cellular mechanisms

**DOI:** 10.3389/fendo.2023.1141337

**Published:** 2023-02-07

**Authors:** Carlos Guillén, Adolfo Garcia-Ocana

**Affiliations:** ^1^ Department of Biochemistry and Molecular Biology, Faculty of Pharmacy, Complutense University, Madrid, Spain; ^2^ Centro de Investigación Biomédica en Red (CIBER) de Diabetes y Enfermedades Metabólicas Asociadas, Instituto de Salud Carlos III, Madrid, Spain; ^3^ Medicine, Endocrinology, Diabetes and Bone Disease, Icahn School of Medicine at Mount Sinai, New Yor, NY, United States

**Keywords:** pancreatic beta (β) cells, type 1 diabetes mellitus, gestational diabetes – mellitus, type 2 (non-insulin-dependent) diabetes mellitus, diabetic patient

Diabetes represents a group of heterogeneous metabolic diseases, which share the appearance of hyperglycemia. The main goal of this Research Topic is to describe different biological processes and molecular mechanisms regulating pancreatic β cell homeostasis with a special emphasis on:

1. Post-translational modifications of different proteins involved in metabolism.
2. Signaling pathways altered during the progression of the disease (mainly in T1DM, T2DM, and gestational diabetes or GDM).
3. Cellular mechanisms that could explain the progression of the disease.
4. Metabolic and epigenetic changes occurring during the progression of diabetes (T1DM, T2DM, and GDM).

This special issue contains nine papers in total: five original research papers presenting novel data about some of the items previously indicated, three mini-reviews, and one systematic review.

In the first of the original papers published in this special issue (Szymczak et al.), the authors report that an enzyme involved in RNA editing, the RNA-specific adenosine deaminase 1 (ADAR1) involved in the transformation of adenine into inosine, plays an important role, favoring a mispairing of adenosine and avoiding an uncontrolled immune response after IFN-γ stimulation. These findings are uncovered using human β cells and human islets. Importantly, ADAR1 is proposed as a novel regulator of pancreatic β cell transcriptome under inflammatory conditions, potentially playing a role in understanding T1DM.

In the second manuscript (Rodrigues et al.), the authors describe a new method for generating a hepatocyte-like cell (HLC) from stem cells. This method is based on the modulation of glucose metabolism and the control of mitochondrial function using insulin, glucose, and dexamethasone. The authors analyze the effect of different concentrations of these agents on the improvement of the hepatic phenotype of these cells, which could serve as a platform for understanding and modeling energy metabolism-related alterations.

In the third paper (Jo et al.), the authors explore the role of posttranslational modification (PTM), called O-GlcNacylation, in different nutrient and stress conditions, in pancreatic β cells. This PTM is controlled by the action of two opposite groups of enzymes, O-GlcNAc transferase (OGT) and O-GlcNAcase (OGA). The findings of this study suggest that a reduction in the action of OGT and the hyperactivity of OGA are deleterious for pancreatic β cells.

In the fourth manuscript of this Research Topic (Oost et al.), the authors describe that magnesium ion, which is commonly defective in T2DM patients, has beneficial effects on adipocytes, increasing the glucose uptake in response to insulin as well as insulin signaling, favoring insulin sensitivity. These results point to the important contribution of this ion in the reduction of insulin resistance in T2DM patients.

In the last original research manuscript (Li et al.), the authors report the effect of several variants of the mitochondrial DNA (mtDNA) associated with diabetes and diabetic kidney disease (DKD) using next-generation sequencing (NGS) from different cohorts of patients. The authors demonstrate a connection between some variants of the mtDNA with either diabetes or DKD, suggesting potential implications on individual therapy for these patients that derives from alterations in mtDNA.

In the first mini-review (García-Aguilar et al.), the authors review the role of polyphenols as a potential treatment for improving pancreatic β cell homeostasis; restoring important cellular processes, such as autophagy; and reducing ER stress and mitochondrial dysfunction, among others. These alterations are commonly observed during the progression of T2DM, suggesting that this group of compounds has therapeutic potential for maintaining healthier pancreatic β cells.

In the second mini-review (Li et al.), the authors review the involvement of circular RNAs (circRNA for short) in the appearance of diabetic foot ulcers (DFU). It has been shown that circRNAs are differentially expressed when diabetic and non-diabetic patients are compared. Therefore, circRNAs could be a potential diagnostic marker and a therapeutic target for treating DFU more effectively.

In the last mini-review (Baumel-Alterzon S et al.), the authors review the role of oxidative stress in the control of one of the master regulators of pancreatic β cell differentiation, Pdx1. It describes the importance of nuclear factor erythroid 2-related factor (Nrf2), as a key molecule involved in the generation of the antioxidant response and the induction of Pdx1, suggesting that pharmacological intervention aimed at modulating Nrf2 and the antioxidant response in pancreatic β cells can lead to the restoration of Pdx1 levels and pancreatic β cell differentiation in diabetes.

In the systematic review by Lewis et al., the authors analyze the changes in the transcriptome and in the miRNAome that could potentially participate in the pathogenesis of gestational diabetes (GDM). This information is valuable for improving the diagnosis, prevention, and treatment of patients with GDM.

## Author contributions

All authors listed have made a substantial, direct, and intellectual contribution to the work and approved it for publication.

